# Pathobiological alterations affecting the distinct clinical courses of pediatric versus adult COVID-19 syndrome

**DOI:** 10.55730/1300-0144.5685

**Published:** 2023-05-31

**Authors:** Emrah ŞENEL, Seyhan TÜRK, Ümit Yavuz MALKAN, Mustafa Çağrı PEKER, Can TÜRK, Hatice Rahmet GÜNER, Gülberk UÇAR, Seval İZDEŞ, Bircan KAYAASLAN, Gülsüm İclal BAYHAN, Serhat EMEKSİZ, İmran HASANOĞLU, Şerife Gökbulut BEKTAŞ, Şeyma BÜTÜN TÜRK, Serhan ÖZCAN, Ahmet ERTÜRK, Ahmet Gökhan AKDAĞ, Ayşegül YILMAZ, İbrahim Celalettin HAZNEDAROĞLU

**Affiliations:** 1 Department of Pediatric Surgery, Surgical Medical Sciences, Faculty of Medicine, Yıldırım Beyazıt University, Ankara City Hospital, Ankara, Turkiye; 2 Department of Biochemistry, Faculty of Pharmacy, Hacettepe University, Ankara, Turkiye; 3 Department of Hematology, Faculty of Medicine, Hacettepe University, Ankara, Turkiye; 4 Department of Economics, Faculty of Economics and Administrative Sciences, Hacettepe University, Ankara, Turkiye; 5 Department of Medical Microbiology, Faculty of Medicine, Lokman Hekim University, Ankara, Turkiye; 6 Department of Infectious Diseases and Clinical Microbiology, Faculty of Medicine, Yıldırım Beyazıt University, Ankara City Hospital, Ankara, Turkiye; 7 Department of Intensive Care Unit, Faculty of Medicine, Yıldırım Beyazıt University, Ankara City Hospital, Ankara, Turkiye; 8 Department of Pediatric Infectious Diseases, Faculty of Medicine, Yıldırım Beyazıt University, Ankara City Hospital, Ankara, Turkiye; 9 Department of Pediatric Intensive Care Unit, Faculty of Medicine, Yıldırım Beyazıt University, Ankara City Hospital, Ankara, Turkiye; 10 Department of Intensive Care Unit, Ankara City Hospital, Ankara, Turkiye; 11 Department of Child Health and Diseases, Ankara City Hospital, Ankara, Turkiye; 12 Department of Child Intensive Care Unit, Kayseri City Training and Research Hospital, Kayseri, Turkiye; 13 Department of Pediatric Surgery, Ankara City Hospital, Ankara, Turkiye

**Keywords:** Pediatric COVID-19, adult COVID-19, *ACE2*, *ANPEP*, *EGFR*, *IGF2R*

## Abstract

**Background/aim:**

The clinical presentation of pediatric coronavirus disease 2019 (COVID-19) is associated with a milder disease course than the adult COVID-19 syndrome. The disease course of COVID-19 has three clinicobiological phases: initiation, propagation, and complication. This study aimed to assess the pathobiological alterations affecting the distinct clinical courses of COVID-19 in pediatric age groups versus the adult population. We hypothesized that critical biogenomic marker expressions drive the mild clinical presentations of pediatric COVID-19.

**Materials and methods:**

Blood samples were obtained from 72 patients with COVID-19 hospitalized at Ankara City Hospital between March and July 2021. Peripheral blood mononuclear cells were isolated using Ficoll-Paque and density-gradient sedimentation. The groups were compared using a t-test and limma analyses. Mean standardized gene expression levels were used to hierarchically cluster genes employing Euclidean Gene Cluster 3.0. The expression levels of identified genes were determined using reverse transcription-polymerase chain reaction.

**Results:**

This study found that *ANPEP* gene expression was significantly downregulated in the pediatric group (p < 0.05, FC: 1.57) and *IGF2R* gene expression was significantly upregulated in the adult group (p < 0.05, FC: 2.98). The study results indicated that the expression of critical biogenomic markers, such as the first-phase (*ACE2* and *ANPEP*) and second-phase (*EGFR* and *IGF2R*) receptor genes, was crucial in the genesis of mild clinical presentations of pediatric COVID-19. *ANPEP* gene expression was lower in pediatric COVID-19.

**Conclusion:**

The interrelationship between the *ANPEP* and *ACE2* genes may prevent the progression of COVID-19 from initiation to the propagating phase in pediatric patients. High *IGF2R* gene expression could potentially contribute to a protective effect and may be a contributing factor for the mild clinical course observed in pediatric patients.

## 1. Introduction

The clinicobiological course of coronavirus disease 2019 (COVID-19) due to the SARS-CoV-2 virus is significantly different in the pediatric population compared to adults [1]. Clinical presentations in adults can be heterogeneous and progressive, ranging from asymptomatic infection or minor upper respiratory tract illness to severe viral pneumonia with respiratory failure [2]. Regarding the severity of the illness, COVID-19 infections can affect multiple organs in adults, such as the respiratory, immunological, hematopoietic, cardiovascular, gastrointestinal, neurological, or musculoskeletal systems [3]. Clinical observations to date point to milder infections and better prognosis in the pediatric population compared to adults [1]. The immune system develops as we grow [4], and it is postulated that children with COVID-19 may have increased levels of inflammatory markers [5]. During the initiation and propagation stages of infection, several renin-angiotensin system (RAS) family genes (e.g., *ACE2* and *ANPEP*) are upregulated, whereas others (e.g., epidermal growth factor receptor - *EGFR* and insulin-like growth factor 2 receptor - *IGF2R*) are downregulated [6,7]. Clinically, the first phase of COVID-19 is the asymptomatic/presymptomatic phase. The *ACE2* and *ANPEP* genes play important roles in this phase. The second clinical phase of COVID-19 is the respiratory phase with mild/moderate/severe symptoms. These are related to the *EGFR* and *IGF2R* genes. The final clinical phase entails multisystemic clinical syndrome with impaired/disproportionate and/or defective immunity, which is associated with immune system genes [6,7]. We reported in the past that lung epithelial cells treated with SARS-CoV-2 revealed high expressions of the *ACE2* and aminopeptidase N (*ANPEP*) genes in the initial phase at 12–24 h. *ACE2* has been found to be an important receptor for the SARS-CoV-2 virus in the initial phase. In addition to *ACE2*, *ANPEP* has also been shown to act as a co-receptor in the first phase and has been suggested to be very important for the virus to develop the infection [6,7]. It seems that *ACE* and *ANPEP* not only affect the attachment of the virus and its entry into the cell but also save time for the virus to perform replication [8]. In addition to changes in the expression of the *ACE2* and *ANPEP* genes, which function as key receptors and co-receptors in the early phase of COVID-19 infection, a decrease in RNA levels is found associated with the *EGFR* and *IGF2R* genes in the propagating phase. This phase is strongly tied to the initial phase and the complicating phase, both of which play key roles in determining the infection’s severity. Toll-like receptors together with *EGFR* are essential for innate immune response generation [9,10]. Therefore, EGFR downregulation may inhibit an early immunological response to an infection. In addition to the *EGFR* transcript, *IGF2R* is also a low-expressed gene during the propagation phase of the viral infection. *IGF2R* encodes insulin-like growth factor 2 and the mannose 6-phosphate receptor. Low *IGF2R* expression is likely a crucial element in the endocytosis-mediated entrance of the virus into the cell [11].

The aim of this study is to assess pathobiological alterations affecting the distinct clinical courses of COVID-19 syndrome in pediatric populations versus adults. Therefore, the hypothesis of our study is that critical biogenomic marker expressions such as the expressions of the first-phase (*ACE2* and *ANPEP*) and second-phase (*EGFR* and *IGF2R*) receptor genes cause the mild clinical presentations of pediatric COVID-19 infections. Elucidation of the physiopathological basis of mild pediatric COVID-19 infection will be helpful for better clinical management of the syndrome in this age group.

## 2. Materials and methods

### 2.1. Blood samples

This research included 72 patients with COVID-19 pneumonia hospitalized at Ankara City Hospital in Ankara, Türkiye, between March and July 2021. The inclusion criteria were as follows: 1) diagnosed with COVID-19 infection; 2) hospitalized at Ankara City Hospital. Patients from whom blood samples could not be taken were excluded from the study. To determine if the expression of the first- and second-phase receptor genes was age-dependent, patients were categorized into pediatric (n = 23) and adult (n = 49) groups. Based on the US Department of Health and Food and Drug Administration’s estimated age ranges for stages of life [12], nine members of our cohort were children (range: 4–11 years, median: 7), 14 were adolescents (range: 14–19 years, median: 16), and 49 were adults (range: 27–91 years, median: 63.5). Information on the codes, ages, groups, and genders of the patients included in the study are given in Supplementary Data File 1. The local ethics committee approved the research protocol with approval number E1-20-1396. Each patient or the patient’s legal guardian signed a written permission form, and tests were conducted in accordance with the WMA Declaration of Helsinki and the Department of Health and Human Services Belmont Report.

### 2.2. Blood collection and PBMC isolation

Up to 10 mL of blood was collected from patients in VACUETTE tubes containing ethylenediamine-tetra acetic acid (EDTA) or in acid citrate dextrose (ACD) tubes. Blood samples were processed immediately. Isolation of peripheral blood mononuclear cells (PBMCs) was performed by density-gradient sedimentation using Ficoll-Paque according to the standard procedures [13].

### 2.3. Expression profiling of genes by q-RT-PCR

In order to determine the expression of the first-phase (*ACE2* and *ANPEP*) and second-phase (*EGFR* and *IGF2R*) receptor genes, RNA extraction, DNA fragmentation, cDNA production, and qPCR experiments were respectively performed. Gene expression was profiled quantitatively using a 7500 Real-Time PCR System and SYBR® Green PCR Master Mix for quantitative reverse transcription-polymerase chain reaction (q-RT-PCR). The PCR procedures were carried out in a cycling environment as recommended by the manufacturer. Every response was checked against *GAPDH* as an endogenous standard. The Primer3 tool (https://primer3.ut.ee/) was used to design the PCR primers (Table 1). The delta-delta Ct method was used to determine gene expression levels [14].

### 2.4. Hierarchical clustering

Genes determined in linear regression analysis were hierarchically clustered with mean standardized gene expression values with the Euclidean Gene Cluster 3.0 program (http://bonsai.hgc.jp/~mdehoon/software/cluster/software.htm) [15]. Data were standardized after cluster analysis, and the standardized data were viewed using TreeView (http://jtreeview.sourceforge.net/) [16]. Hierarchical clustering was performed by clustering both genes and arrays using Euclidian distance as a similarity metric and complete linkage as a clustering method.

### 2.5. Statistical analyses

To determine if there was a statistically significant difference between the expression of the relevant genes in the pediatric population versus adults, we performed a t-test and limma analysis [17]. The limma package is a core component of Bioconductor, an R-based open-source software development project for statistical genomics. It has proven a popular choice for the analysis of data from experiments involving microarrays, PCR, protein arrays, and other platforms. It contains rich features for handling complex experimental designs and for information-borrowing to overcome the problem of small sample sizes [17]. We also performed the Pearson product-moment correlation coefficient test to describe the strength and direction of the linear relationships between the relevant gene expressions and age. Additionally, we performed post hoc power analysis using G*Power software version 3.1.9.7 to determine the statistical power of the study based on the observed effect size and sample size.

## 3. Results

**Figure 1:**
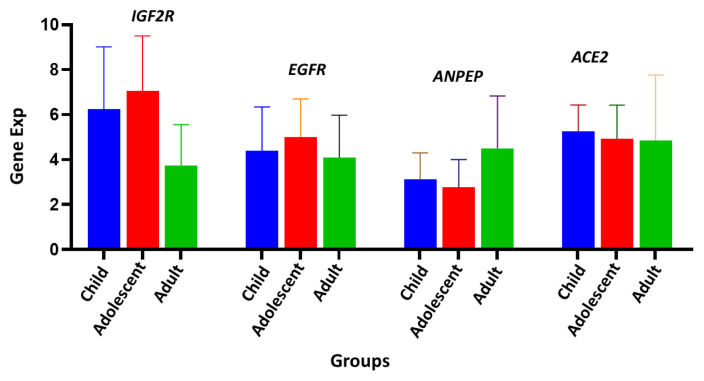
Gene expression analysis of first- and second-phase biomarker genes. *IGF2R*, *EGFR*, *ANPEP*, and *ACE2* were respectively quantified by real-time qPCR in child, adolescent, and adult groups. *GAPDH* was used as an endogenous control.

**Figure 2:**
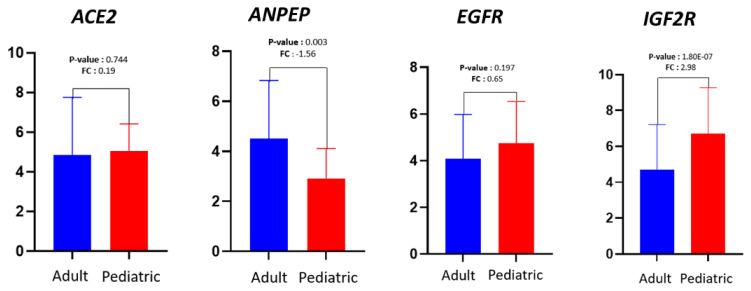
Comparison of first- and second-phase biomarker genes between adult and pediatric groups. The p-values indicate t-test comparisons of expression levels between adult and pediatric groups. FC values describe the change in the expression level of each gene.

**Figure 3:**
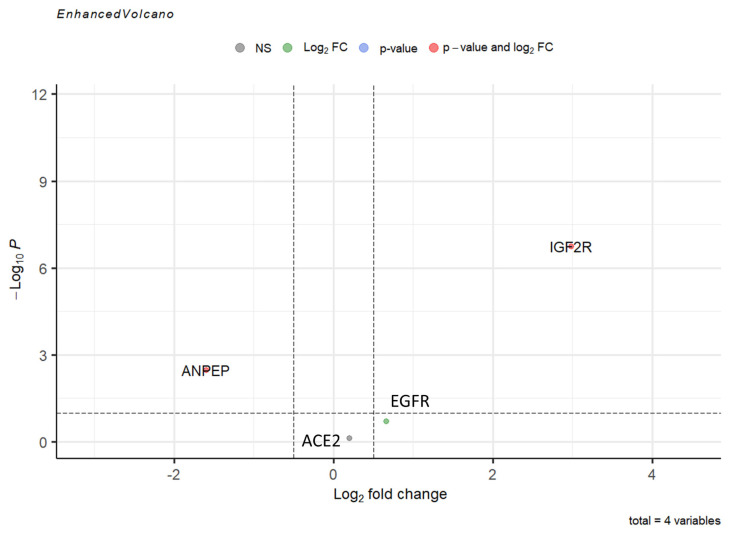
Comparison of average expression levels of first- and second-phase biomarker genes. The limma analysis showed that, among the four genes, two (*ANPEP* and *IGF2R*) were expressed statistically significantly differently between the groups.

**Figure 4:**
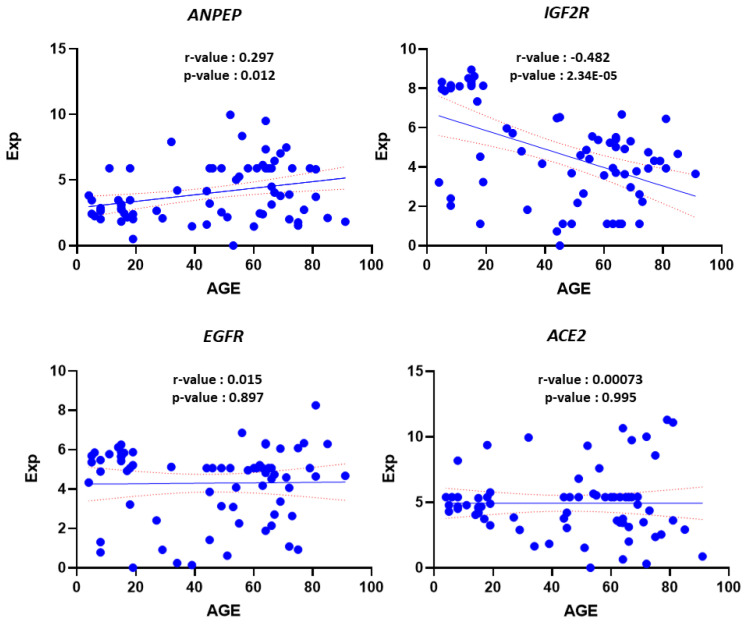
Linear correlation analysis of gene expression values with age. Analysis showed that *ANPEP* and *IGF2R* correlated significantly with age.

**Figure 5:**
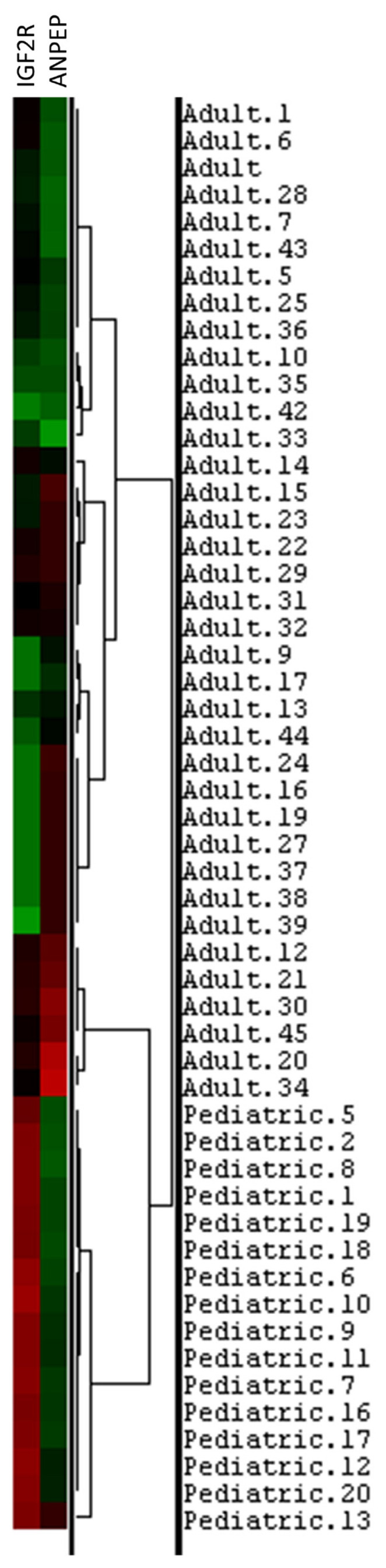
Hierarchical analysis confirmed that *IGF2R* and *ANPEP* can distinguish between adult and pediatric groups well.

Polymorphonuclear leukocyte (PMNL) cells were isolated from 70 out of the total collected 72 blood samples from COVID-19 patients (Table 2). Our sample population was divided into two groups of pediatric and adult patients. Infancy, childhood, and adolescence were the subgroups of the pediatric age group. The frequency and percentage of each gene within these groups are presented in Table 3. In terms of gender distribution, there were 15 male and 8 female patients in the pediatric group, while there were 32 male and 16 female patients in the adult group (Table 3). Approximately 1–2 × 10^7^ PMNL cells were isolated from each patient’s blood sample. Approximately 50–100 ng of RNA was extracted from each sample and used for RT-PCR to determine the level of gene expression. Six cases with active bacterial infection were excluded from analysis since active bacterial infection can alter immunogenomic features. Figure 1 depicts the expression levels of each gene in the different groups. To determine whether there was a statistically significant difference in the expression levels of genes (*ACE2*, *ANPEP*, *EGFR*, and *IGF2R*) between the groups, the average expression values of the genes were compared using the t-test and limma statistical results (Figure 2). According to the results of the Student two-tailed t-test, while the *ANPEP* gene was significantly downregulated in the pediatric group (p < 0.05, FC: 1.57), the *IGF2R* gene was significantly upregulated in the adult group (p < 0.05, FC: 2.98). *EGFR* and *IGF2R* expression levels were not significantly different between the adult and pediatric groups (p > 0.05). In addition, limma analysis results demonstrated that two genes (*ANPEP* and *IGF2R*) were expressed differently in the pediatric and adult groups (Figure 3). Post hoc power analysis showed that the study achieved statistical power of 0.87 with an effect size of 0.8 and sample sizes of 23 for the pediatric group and 49 for the adult group, with a significance level of p < 0.05. Comparing the male and female samples from different groups revealed no statistically significant differences in the expressions of the genes (data not shown). The results of Pearson correlation analysis revealed that the *ANPEP* gene had a significant direct relationship with age, whereas the *IGF2R* gene had a significant inverse relationship with age. Neither *ACE2* nor *EGFR* demonstrated a significant correlation with age (Figure 4). Hierarchical clustering analysis showed that the *ANPEP* and *IGF2R* genes could distinguish between pediatric and adult patients very well after the exclusion of samples with expression values beyond the mean expression values of the groups (Figure 5).

## 4. Discussion

The results of the current study support the hypothesis that the mild clinical presentations of pediatric COVID-19 are driven by the expression of critical biogenomic markers, specifically the first-phase receptor genes (*ACE2* and *ANPEP*) and second-phase receptor genes (*EGFR* and *IGF2R*). In a previous study, our group described the three clinicobiological phases (initiation, propagation, and complication phases) of human SARS-associated coronavirus infections [6]. The interferon (IFN) family plays a crucial role in the impact of SARS-CoV-2 [18]. We previously demonstrated that important *IFN* gene expressions are altered upon exposure to the SARS-CoV-2 virus [18]. One of the primary mechanisms for early infection control is the detection of continuously exposed pathogens, which stimulates the immediate replenishment of innate immune cells from the early stages of their development through emergency hematopoiesis [19]. However, the functional success of this mechanism can become dangerous when excessive stimulating signals accelerate immunological catastrophes. Metabolic or hyperinflammatory conditions in aging exacerbate this mechanism and lead to uncontrolled emergency myelopoiesis, resulting in severe COVID-19 disease [19]. This mechanism may explain the different clinical courses observed in pediatric and adult COVID-19 cases. Furthermore, adult COVID-19 patients with hypertension may more frequently and easily progress from the propagation phase to the complication phase due to alterations in the *EGFR* and *IGF2R* genes [7]. The higher incidence of hypertension in adult patients may also contribute to the differences in clinical course observed in COVID-19 between pediatric and adult populations. Gastrointestinal manifestations of COVID-19 can present as acute abdomen, which may be caused by conditions such as acute pancreatitis, acute appendicitis, intestinal obstruction, bowel ischemia, hemoperitoneum, or abdominal compartment syndrome [20]. The involvement of the gastrointestinal system in COVID-19 can be attributed to direct viral injury and/or an inflammatory immune response. This involvement can lead to malabsorption, an imbalance in intestinal secretions and gut mucosal integrity, and stimulation of the enteric nervous system [20].

Additionally, the *ANPEP* gene (also known as *CD13*) has been associated with gastrointestinal system involvement in COVID-19 [6]. *ANPEP* and *ACE* are two genes that have been implicated in COVID-19 due to their involvement in the renin-angiotensin-aldosterone system (RAS). The RAS is a complex hormonal pathway that regulates blood pressure, fluid balance, and electrolyte homeostasis. ACE is a key enzyme in this system, converting angiotensin I to angiotensin II, a potent vasoconstrictor that promotes inflammation [21]. The *ANPEP* and *ACE* genes are also known to participate in other important pathways. *ACE*, for instance, is involved in the ACE inhibitor pathway, early SARS-CoV-2 infection events, and peptide hormone metabolism [22–24]. On the other hand, *ANPEP* is involved in the innate immune system, glutathione conjugation, and protein metabolism pathways [25–27]. The SARS-CoV-2 virus, responsible for causing COVID-19, utilizes the *ACE2* receptor to enter human cells. *ACE2* is expressed in different tissues of the body, including the lungs. Recent studies have indicated that *ANPEP*, which codes for CD13, may also play a role in the viral entry process. In certain cell types, *ANPEP* can function as an alternative or co-receptor for the virus [28].

In the current study, we observed that the expression of the *ANPEP* gene was lower in pediatric patients with COVID-19. This lower expression may have a protective effect in the pediatric age group, as the *ANPEP* gene is involved in the intestinal penetration of SARS-CoV-2 [7]. Moreover, the dysregulation of the sodium-dependent glucose transporter (SGLT1 or SLC5A1) in the intestinal epithelium, controlled by *ACE2*, has been linked to the pathogenesis of diabetes mellitus, which may contribute to the higher mortality observed in COVID-19 patients with diabetes [29].

The high expression of *ACE2* in mucosal cells of the intestine suggests that they could serve as potential entry sites for the virus and support its replication [29].

The interrelationship between the *ANPEP* and *ACE* genes might impede the progression of COVID-19 from the initiation phase to the propagating phase in pediatric patients. Notably, both low *ANPEP* gene expression levels and mild clinical disease courses were common features in pediatric COVID-19 patients and in adult patients without gastrointestinal involvement. Thus, the *ACE* and *ANPEP* genes may contribute to the severity and progression of COVID-19 by influencing the virus’s ability to enter human cells and modulating the response of the RAS to the infection.

Understanding the genetic factors that influence the susceptibility and severity of COVID-19 could help identify individuals at higher risk and guide the development of new therapeutic approaches. The present study has revealed that the expression of the *IGF2R* gene is higher in pediatric patients with COVID-19. Previous research demonstrated that with increasing exposure time to SARS-CoV-2, two receptors, *EGFR* and *IGF2R*, which play crucial roles in the RAS signaling pathway, are significantly downregulated in infected human bronchial epithelial cells during the propagating phase of COVID-19 [7].

*EGFR* is a gene involved in cell growth and proliferation. It has been found to be upregulated in lung cells infected with SARS-CoV-2. This upregulation may contribute to the development of lung inflammation and the lung damage observed in severe cases of COVID-19. Furthermore, certain studies suggest that drugs targeting *EGFR* may hold potential as treatments for COVID-19, although further research is required to explore this possibility [30]. It is important to note that while the roles of *EGFR* and *IGF2R* in the context of COVID-19 have been investigated, their precise significance in the disease is still being explored. Ongoing research aims to provide a better understanding of the involvement of these genes in COVID-19 and their potential as therapeutic targets. *IGF2R* is a gene that regulates the activity of insulin-like growth factors, which are involved in cell growth and division. In lung cells infected with SARS-CoV-2, *IGF2R* has been found to be downregulated, and this downregulation may contribute to the development of lung inflammation and damage observed in severe cases of COVID-19 [7]. Additionally, some studies have suggested that *IGF2R* may play a role in the immune response to viral infections, although further research is necessary to fully understand this role [31].

Furthermore, insulin-like growth factor 2 (IGF2) has been found to be decreased in the serum of individuals with diabetes [32]. Diabetic patients generally exhibit a poorer prognosis in COVID-19, as diabetes is considered a risk factor for adverse outcomes [33]. In light of these data, the high expression of the *IGF2R* gene in pediatric patients may have a protective role and could contribute to the milder clinical course of COVID-19 observed in this population. The interrelationship between the *EGFR* and *IGF2R* genes might impede the progression of COVID-19 from the propagating phase to the complicating phase in pediatric patients.

Overall, although the *EGFR* and *IGF2R* genes have been implicated in COVID-19, further research is required to fully comprehend their significance in the disease and to identify potential targets for treatments.

According to our previous study, COVID-19 exhibits three distinct phases: the initiating, propagating, and complicating phases. Each phase is associated with specific genes: *ACE2* and *ANPEP* in the initiating phase, *EGFR* and *IGF2R* in the propagating phase, and immune system-related genes in the complicating phase [6].

Generally, the clinical course of COVID-19 tends to be more severe in adults compared to pediatric patients. In the initiating phase and in some cases in the propagating phase, COVID-19 treatment can be managed in outpatient clinic settings. However, if patients progress to the complicating phase, their clinical condition deteriorates, necessitating hospitalization and, in some cases, admission to intensive care units. One possible explanation for the divergent clinical courses observed between adult and pediatric COVID-19 patients is alterations in the *ANPEP* and *IGF2R* genes. Particularly in adult patients, screening for *ANPEP* and *IGF2R* gene expressions could be valuable for predicting the severity of the clinical course of COVID-19. This predictive capability may have implications for future treatment algorithms in COVID-19.

It is important to note that further research and investigation are required to fully understand the roles of these genes and their potential implications for COVID-19 treatment and management. Our research group has proposed a model suggesting that the incorporation of the SARS-CoV-2 virus into the human virobiota occurs through three distinct pathobiological phases: induction, consolidation, and maintenance [34].

This chimerism process involves the integration of the SARS-CoV-2 virus into the human genome, particularly within the intestinal virobiota [34]. Additionally, it has been observed that the Omicron variant of the virus possesses unique characteristics that enhance chimerism-mediated immunotherapy without causing high mortality rates by evading interferon gene expression [35].

The focus of this paper has been exploring the reasons behind the divergent clinical courses observed in pediatric and adult COVID-19 patients. We have clinically validated our previous hypothesis regarding the three critical clinicobiological phases of human SARS-associated coronavirus infections [6], along with their corresponding genes for each phase. Furthermore, we have shown that COVID-19 induces differential gene expression patterns in adult and pediatric patients, which may contribute to the observed differences in clinical course between these two age groups. Specifically, we have emphasized the significance of the *ANPEP* and *IGF2R* genes, which play unique roles in the progression of COVID-19 from the initiating phase to the propagating phase.

The findings presented in this study can serve as a foundation for future investigations focusing on the clinical implications of distinct gene expression patterns in COVID-19. One important limitation of this study is the relatively small number of study participants, which was due to corruption of the samples. The limited sample size might have had an impact on the generalizability and statistical power of the findings. It is crucial to replicate and validate the results of this study using larger sample sizes in future studies. With the inclusion of a larger number of participants, the findings would be more robust and reliable, providing a stronger basis for drawing conclusions and identifying clinical implications.

It is also worth noting that additional factors, such as demographic characteristics, comorbidities, and environmental factors, could potentially influence the clinical course of COVID-19. Controlling for these factors and conducting further investigations with larger cohorts would provide a more comprehensive understanding of the relationship between gene expression patterns and the clinical outcomes of COVID-19.

In conclusion, the expression of critical biogenomic markers, including the *ACE2*, *ANPEP*, *EGFR*, and *IGF2R* genes, plays a significant role in the development of mild clinical presentations in pediatric COVID-19 cases. Pediatric patients exhibit lower *ANPEP* gene expressions, which may contribute to their milder clinical courses. The interplay between the *ANPEP* and *ACE* genes could potentially prevent the progression of COVID-19 from the initiation phase to the propagating phase in pediatric patients. Furthermore, the higher expression of the *IGF2R* gene in pediatric patients may confer a protective effect and contribute to the mild clinical course observed in this age group. The interrelationship between *EGFR* and *IGF2R* might also play a role in impeding the progression of COVID-19 from the propagating phase to the complicating phase in pediatric patients.

However, further research with larger sample sizes is needed to validate these findings and better understand the complex genetic mechanisms underlying the clinical course of COVID-19 in pediatric patients. Exploring the interplay of these genes could have implications for predicting disease severity and guiding future treatment strategies for pediatric COVID-19 cases.

## Supplementary Information



## Figures and Tables

**Table 1:** Primer sequences for first- and second-phases genes.

Symbol	Forward	Reverse
*ACE2*	TGGGTCTTCAGTGCTCTCAG	ACCCCACATATCACCAAGCA
*ANPEP*	GGCCCCATGAAGAACTACCT	TCAGCCTCATTGACCAGTGT
*GAPDH*	CCAGAACATCATCCCTGCCT	CCTGCTTCACCACCTTCTTG
*EGFR*	TCATGCTCTACAACCCCACC	GCACTTCTTACACTTGCGGA
*IGF2R*	CTTTGACAGCGAGAATCCCG	TCACTGTTTCCCTCCTCTCC

**Table 2:** Descriptive statistics: number of valid and missing samples for each gene, respectively.

		*ACE2*	*ANPEP*	*EGFR*	*IGF2R*
N	Valid	70	70	69	69
	Missing	2	2	3	3

**Table 3:** Frequency and percent analysis of valid and missing samples belonging to different age groups.

		Frequency	Percent	Valid percent	Cumulative percent
*ACE2* Valid	Infancy	0	0%	0%	0%
	Childhood	9	12.85%	12.85%	12.85%
	Adolescence	13	18.57%	18.57%	18.57%
	Adult	48	68.57%	68.57%	68.57%
	Total	70	100%	100%	100%
*ANPEP* Valid	Infancy	0	0%	0%	0%
	Childhood	9	12.85%	12.85%	12.85%
	Adolescence	13	18.57%	18.57%	18.57%
	Adult	48	68.57%	68.57%	68.57%
	Total	70	100%	100%	100%
*EGFR* Valid	Infancy	0	0%	0%	0%
	Childhood	9	12.85%	12.85%	12.85%
	Adolescence	12	17.39%	17.39%	17.39%
	Adult	48	68.57%	68.57%	68.57%
	Total	69	99%	99%	99%
*IGF2R* Valid	Infancy	0	0%	0%	0%
	Childhood	9	12.85%	12.85%	12.85%
	Adolescence	13	18.84%	18.84%	18.84%
	Adult	47	68.11%	68.11%	68.11%
	Total	69	99%	99%	99%
